# Machine learning identifies differences between breast milk and formula in the gut microbiome

**DOI:** 10.1017/gmb.2026.10020

**Published:** 2026-05-08

**Authors:** Ting Chia Liu, David Rojas-Velazquez, Sarah Kidwai, Astrid Hogenkamp, Johan Garssen, Aletta D. Kraneveld, Alejandro Lopez-Rincon

**Affiliations:** 1 Division of Pharmacology, Utrecht Institute for Pharmaceutical Sciences, Faculty of Science, Utrecht, The Netherlands; 2 Department of Data Science, Julius Center for Health Sciences and Primary Care, University Medical Center Utrecht, Utrecht, The Netherlands; 3 Danone Global Research & Innovation Center, Utrecht, The Netherlands; 4 Department of Neuroscience, Faculty of Science, Vrije Universiteit Amsterdam, Amsterdam, The Netherlands

**Keywords:** feature selection, machine learning, biomarker discovery, deep learning

## Abstract

In this study, we analysed differences in the infant gut microbiome between breastfed and formula-fed infants using novel machine learning techniques. Breast milk, rich in bioactive agents, supports microbiota composition and immune development, while formulas aim to replicate its nutritional profile. We applied a methodology combining the DADA2 pipeline for 16S rRNA sequencing with the Recursive Ensemble Feature Selection (REFS) algorithm for biomarker discovery. We analysed three publicly available 16S rRNA datasets: PRJNA633365 (70 stool samples from China), PRJDB7295 (40 stool samples from the Philippines), and PRJNA562650 (40 stool samples from China). The discovery dataset (PRJNA633365) revealed 16 significant taxa out of 1,227, validated across the other two datasets. Next, we compared REFS performance with another feature selection algorithm, SelectKBest. Finally, we conducted a literature review to explore links between identified taxa and medical conditions. Additionally, we used MicrobiomeAnalyst to examine associations with diseases, diet, and lifestyle. Our results show differences in the bacterial composition between breastfed and formula-fed infants, and these findings were validated in two independent datasets. Future research should explore the functional roles of these taxa and consider regional and dietary variability to enhance understanding of microbiome dynamics and long-term health outcomes.

## Introduction

In early life, the human microbiome plays a pivotal role in shaping immune system development (Reynolds and Bettini, 2023). The composition of the microbes depends heavily on early nutrition, and variations in gut bacteria of breastfed versus formula-fed infants have been observed and published frequently. Breast milk is considered the best source of nutrition for babies (Martin *et al.*, 2016). It contains bioactive agents that support the development of the gastrointestinal tract, including the microbiota composition, the immune system, as well as brain development (Martin *et al.*, 2016). When an infant cannot be (fully) breastfed, it is required to explore alternative options such as infant milk formulas. Infant milk formulas are designed to be an effective alternative to breast milk, aiming to replicate its nutritional profile for the growth and development of the infant. Formulas are generally based on cow’s milk or soy milk and are often supplemented with additional ingredients such as prebiotic oligosaccharides, micronutrients, and specific fat blends which include essential fatty acids such as arachidonic acid and docosahexaenoic acid (Martin *et al.*, 2016).

The effect of breast milk and infant milk formula on the composition of the infant’s microbiome has been compared in various studies (Brink *et al.*, 2020; Chong *et al.*, 2022; Ho *et al.*, 2018; Martin *et al.*, 2016; Roggero *et al.*, 2020; Wang *et al.*, 2020; Yao *et al.*, 2021). Such studies often aim to find associations between the microbiome and health-related outcomes such as infant body composition and development (Goran *et al.*, 2017; Laursen *et al.*, 2021; Ronan *et al.*, 2021), various medical implications in infants such as allergies (Akagawa and Kaneko, 2022; Björkstén *et al.*, 2001; Francavilla *et al.*, 2012; Hendrickx *et al.*, 2023; Hoskinson *et al.*, 2023; Jin *et al.*, 2023; Ling *et al.*, 2014; Marrs *et al.*, 2021; Mennini *et al.*, 2021; Savage *et al.*, 2018; Xu *et al.*, 2025), or asthma (Friedman and Zeiger, 2005; Oddy, 2004). All of these studies are focused on identifying potentially beneficial bacteria that can be included in infant formulas to improve their effectiveness for the growth and development of the infant and become a supplement increasingly similar to breast milk. Despite known differences in feeding practices, there remains a gap in understanding the scope of how early feeding influences microbiome development over time and how differences can affect long-term health outcomes. This is complicated by the large volume and complexity of microbiome data, which is difficult to analyze using traditional statistical methods.

Biomarker discovery using machine learning is an area of growing relevance in the medical field. The main objective is to identify potential biomarkers for disease diagnosis by analyzing omics data, such as the human microbiome. While this approach is promising, reproducibility and validation in independent datasets remain significant challenges. Recent studies propose methodologies that aim to mitigate these limitations and provide reliable and robust results (Rojas-Velazquez *et al.*, 2024b, 2025).

In the current study, we applied a reproducible methodology for biomarker discovery that analyzes the human microbiome (16S rRNA sequences) with the aim of identifying bacterial taxa that differ between breastfed and formula-fed infants and analysing their relationship with medical conditions such as cow’s milk allergy. Developing a reproducible methodology for analysing 16S rRNA sequencing data is essential to ensure consistency and reliability across studies. Microbiome datasets are complex and prone to variability due to sequencing errors, batch effects, and differences in processing pipelines (Loganathan and Priya Doss, 2022).

An approach enabling accurate taxonomic classification, proper normalization of compositional data, and meaningful biological interpretation is required to allow researchers to compare results across studies and apply findings to biomedical research (Rojas-Velazquez *et al.*, 2024b, 2025). Therefore, the methodology applied in this work was previously developed by our research group; it combines a DADA2-based script for high-quality sequence processing with the Recursive Ensemble Feature Selection (REFS) algorithm for biomarker discovery, providing a robust framework for microbiome analysis (Rojas-Velazquez *et al.*, 2024b).

We previously applied this methodology in different studies involving the analysis of human microbiome associated with diseases such as Inflammatory Bowel Disease, Type-2 Diabetes, Autism Spectrum Disorder (Rojas-Velazquez *et al.*, 2024b), and Parkinson’s Disease (Rojas-Velazquez *et al.*, 2025). Similarly, we have applied our methodologies to analyze other types of omics data, including mRNA and MiRNA (Benner *et al.*, 2021; Blankestijn *et al.*, 2023; Kamphorst *et al.*, 2023; Lopez-Rincon *et al.*, 2019, 2020; Metselaar *et al.*, 2021; Rojas-Velazquez *et al.*, 2023), where adjustments are only made to the data processing phase according to the type of data analyzed; the feature selection (biomarker discovery) and validation phases remain unchanged.

In addition, we made a performance comparison between REFS and another feature selection algorithm called SelectKbest. This means that in the feature selection phase (biomarker discovery), we exchange the REFS algorithm for SelectKbest, leaving the other phases of the methodology unchanged. We perform a literature analysis to identify the relationship between the identified bacterial taxa and medical conditions. Next, we used the web-based tool called MicrobiomeAnalyst (Dhariwal *et al.*, 2017) to analyze the relationship between the identified bacterial taxa and different medical conditions, diet, and lifestyle.

## Methods

We applied a methodology previously developed in our research group (Rojas-Velazquez *et al.*, 2024b). This methodology combines a DADA2-based script and the Recursive Ensemble Feature Selection (REFS) algorithm (Open source tools available on GitHub, https://github.com/steppenwolf0/MicrobiomeREFS). DADA2 is an open-source R package designed to enhance the analysis of 16S rRNA sequences: quality filtering, dereplication, sequence variant inference, chimera detection, and merging paired-end reads (Callahan *et al.*, 2016). REFS is a reliable and effective method for discovering biomarkers that distinguish between groups (e.g., cases/controls) in datasets by achieving the highest accuracy with a minimal number of features (Benner *et al.*, 2021; Blankestijn *et al.*, 2023; Kamphorst *et al.*, 2023; Lopez-Rincon *et al.*, 2019, 2020; Metselaar *et al.*, 2021; Rojas-Velazquez *et al.*, 2023, 2024a, 2025). REFS is an ensemble composed of eight algorithms from the scikit-learn (Pedregosa *et al.*, 2011) that selects a reduced set of features based on the accuracy in differentiating between case and control groups using the smallest number of features using a nested-based 10-fold cross-validation approach (Lopez-Rincon *et al.*, 2019). The methodology consists of four phases, check (Rojas-Velazquez *et al.*, 2024b) for detailed information:

1. *Dataset selection criteria*: involves selecting 16S rRNA amplicon sequencing datasets from a common domain (e.g., disease, disorder, or medication) that include at least two labeled groups (e.g., control and case) with a minimum of 10 samples each, consistent sample sources (e.g., faeces or tissue), and well-documented metadata specifying sample group assignments.
2. *Raw data processing*: uses an R script based on the DADA2 pipeline (DADA2 pipeline is available in https://benjjneb.github.io/dada2/tutorial_1_8.html) to perform the amplicon workflow on 16S rRNA sequences to generate amplicon sequence variants (ASVs) from the selected datasets. The taxonomy assignment was performed based on the SILVA_SSU_r138_2019 (available in: http://www2.decipher.codes/Classification/TrainingSets/) reference database. In addition to using SILVA for taxonomy assignment, we use the online tool BLAST to complement the taxonomy assigned by the DADA2 process. BLAST (https://support.nlm.nih.gov/kbArticle/?pn=KA-05205) is an NCBI tool that compares biological sequences to reference databases to find similar matches and identify organisms at the highest taxonomic level possible; for example, it is used to classify bacteria by matching their 16S rRNA sequences to known taxa. For this task, we selected the option *blastn* in the Nucleotide BLAST suite; the reference dataset was the *Core nucleotide database (core_nt). The assignment at the genus level was made taking into account the highest number of hits at that level for that genus name and the number of matches suggested for that level.*
3. *Feature selection*: applying REFS to identify unique sequence-based features from a discovery dataset, chosen for having the shortest sequences after processing according to established in Rojas-Velazquez *et al.* (2024b), followed by a validation module composed by five different classifiers to assess performance via Area Under the Receiver Operating Characteristic Curve (AUC-ROC), all repeated at least 10 times to reduce stochasticity from certain classifiers (e.g., Random Forest) and the internal cross-validation.
4. *Testing*: involves evaluating the features selected by REFS in at least two separate datasets by searching each feature in the testing datasets, summing its abundance if it appears multiple times, and then validating these features using the validation module composed of five algorithms – run once per dataset with sample labels, features found, and feature abundances – to measure diagnostic accuracy using AUC-ROC.

In addition, we compared the performance of our methodology against another feature selection algorithm called SelectKbest. This algorithm is a feature selection method that picks the top *k* features based on a scoring function in which the top *k* features with the highest scores are selected (Pedregosa *et al.*, 2011). For this performance comparison, we replaced REFS with SelectKbest in the feature selection phase. We set k = 16 because that was the number of taxa selected by REFS. Finally, we applied the validation module to compare Area under the Receiver Operating Characteristic Curve (AUC-ROC) between both algorithms.

The identified bacterial taxa were analyzed using the Taxon Set Analysis tool to identify if they are related to different diseases and symptoms. This tool belongs to MicrobiomeAnalyst, which is a web-based platform for comprehensive analysis of microbiome data (Dhariwal *et al.*, 2017). Although this tool is not part of the methodology (Rojas-Velazquez *et al.*, 2024b), the results obtained provide valuable complementary information to our literature analysis. It is important to mention that the use of MicrobiomeAnalyst focuses on the analysis of bacterial taxa in relation to external factors such as diet and lifestyle. We use it specifically to explore the associations with breast milk or infant formula. We do not intend to directly analyse the diet of mothers or infants. For these analyses, we select the taxon set analysis option, where we enter a list of taxa at different levels using the mixed-level taxon names option. After uploading the files, the analysis of the entered information will be displayed. Click the Submit button, and in the new window, select host-intrinsic taxon sets to find the taxa–disease relationship. For the diet and lifestyle analysis, repeat the same steps: upload the list of taxa and then select the host-diet taxon sets option.

**Figure 1.**
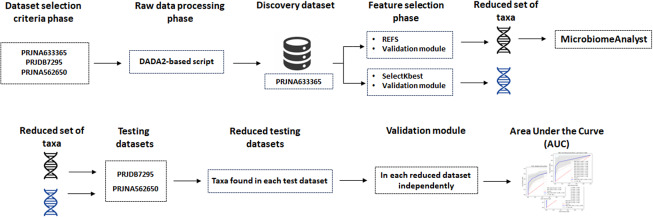
Overview of the methodology followed in this research work. The upper section, from left to right, shows the dataset selection criteria, raw data processing, and feature selection phases, including the analysis conducted using MicrobiomeAnalyst and the SelectKbest experiment. The lower section corresponds to the testing phase.

The workflow followed in this work is shown in Figure 1, where the upper section, from left to right, shows the dataset selection criteria phase where the three datasets were selected according to the criteria established in Rojas-Velazquez *et al.* (2024b), the raw data processing phase using a DADA2-based script to generate ASVs that will be the input data for the next phase, the feature selection phase using the REFS algorithm and at the same time the SelectKbest algorithm to identify the reduced set of features and for the performance comparison between both approaches, the analysis performed using MicrobiomeAnalyst is included as an additional module. The lower section, from left to right, represents the searching of the reduced set of features in the testing datasets. Once the features are found on each testing dataset, we execute the validation module (independently on each testing dataset) on the taxa found on each dataset to obtain, as a result, the AUC-ROC. At the same time, the resulting AUC-ROC is going to be the metric for the performance comparison between REFS and SelectKbest.

### Dataset selection criteria

Following the guidelines established in Rojas-Velazquez *et al.* (2024b) for the *dataset selection criteria* phase, we selected the following datasets from the NCBI (https://www.ncbi.nlm.nih.gov/) repository:

1. **PRJNA633365 (**Ma *et al.*, 2020 **):** contains 16S rRNA sequencing data collected from 238 faecal samples, which were collected from infants in China (33 female and 32 male) at three time points after birth: 40 days, 3 months, and 6 months, the baby delivery mode ratio (vaginal delivery/caesarean section delivery) is 1.1 for breast-fed, 0.6 formula A and 0.7 formula B. For this dataset, we considered the final time period (6 months), and we merged the groups *formula A* and *formula B* into one infant formula group. After filtering, we only used 70 samples: 22 samples for breast milk and 43 samples for formula. We identify six damaged/poor quality samples that were discarded (SRR11804035, SRR11804058, SRR11804165, SRR11804192, SRR11804231, and SRR11804235).
2. **PRJDB7295** (https://www.ncbi.nlm.nih.gov/sra/?term=PRJDB7295)**:** contains 16S rRNA sequencing data collected from 60 faecal samples, which were collected from infants in the Philippines (22 female and 18 male) at three time points after birth: 2 months, 3 months, and 4 months. According to the metadata, the delivery mode (vaginal delivery/caesarean section delivery) proportion is 22 caesarean section deliveries and 38 vaginal deliveries. For this dataset, we considered 40 samples from all periods and only breast milk and infant formula: 24 samples for breast milk and 16 samples for infant formula.
3. **PRJNA562650 (**Li *et al.*, 2020 **):** contains 16S rRNA sequencing data collected from 77 faecal samples, which were collected from infants in China (no additional information) at different time points after birth, from 5.1 weeks to 40.3 weeks. The delivery mode (vaginal delivery/caesarean section delivery) proportion is 44 caesarean section deliveries and 33 vaginal deliveries. We considered only 40 samples from all periods: 26 samples for breast milk and 14 samples for infant formula.

We selected a minimum of three datasets (one for discovery and two for testing) due to the challenges of working with datasets that contain omics data, such as the availability of public datasets, inconsistent sample sources, poor sequence quality, insufficient documentation, and batch effects caused by variations in sequencing equipment (Rojas-Velazquez *et al.*, 2024b). The criterion used to select the analyzed samples for PRJNA633365 was that infants typically begin a solid diet between 4 and 6 months of age, which influences the composition of the gut microbiota and may be an important factor in the development of metabolic problems in adulthood (Ding *et al.*, 2025; Kuo *et al.*, 2011). For the remaining two datasets, we selected samples from all periods to meet the minimum number of samples established in Rojas-Velazquez *et al.* (2024b).

## Results

### Raw data processing phase

After applying the amplicon workflow (filtering, trimming, and taxonomy assignment) to the raw sequencing data from the three datasets using DADA2, we obtained the corresponding ASVs. Table 1 summarizes the technical specifications of each dataset: the 16S rRNA target regions, the characteristics of the raw reads, the trimming/truncation parameters applied, the lengths of the resulting ASVs, and the total number of ASVs generated for each dataset. The trimming parameter applied for PRJDB7295 means that all reads are trimmed of the first 10 bp (trimLeft = 10), forward reads are truncated at 250 bp, and reverse reads are truncated at 220 bp (truncLen = c(250,220)).

**Table 1.** Summary of datasets characteristics: NCBI accession numbers, targeted 16S rRNA gene regions, approximate raw read lengths, final ASV lengths resulting from DADA2 processing, trimming/truncation parameters, and the total number of ASVs generated on each dataset.

Accession number	Target region	Raw reads length	ASV length	Trimming parameters	ASVs generated
PRJNA633365*	V4	~200 bp (R1/R2)	~263 bp	none	1227
PRJDB7295	V3-V4	~300 bd (R1/R2)	~415 bp	trimLeft = 10 truncLen = c(250,220)	1799
PRJNA562650	V3-V4	~241 bp (R1/R2)	~456 bp	none	2385

* Discovery dataset.

Because our methodology focuses on finding a signature (a sequence contained within another) that is present in other databases to validate its effectiveness in distinguishing between groups (cases versus controls) through classification algorithms, we selected PRJNA633365 as the discovery dataset based on the *eligibility criteria* which states: “the discovery dataset is the one that contains the shortest sequence length after the raw data processing phase” (Rojas-Velazquez *et al.*, 2024b). It is important to note that PRJNA633365 16S rRNA V4, while PRJDB7295 and PRJNA562650 are V3-V4. This means that any feature found in PRJNA633365 can be potentially identified in the V4 region of the other two datasets, making the length criterion secondary compared to the region they share. PRJDB7295 and PRJNA562650 were selected as testing datasets to validate the set of features identified in the discovery phase.

### Feature selection

**Figure 2.**
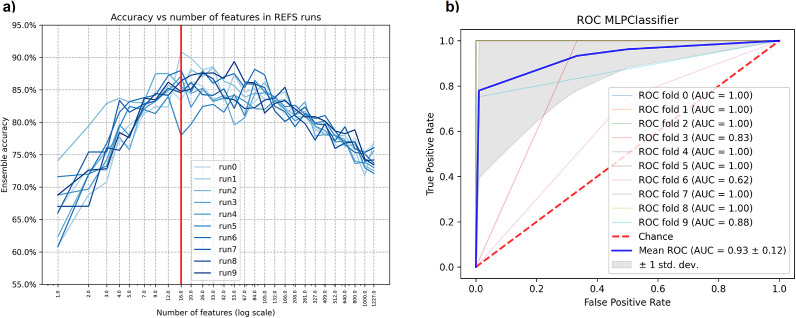
(A) Feature selection phase: the reduced set of features that achieves the highest accuracy is identified by the red line; (B) AUC-ROC for the classifier with the best performance in the validation module applied to the discovery dataset PRJNA633365.

After running REFS on the discovery dataset, the resulting reduced set of features contained 16 out of the original 1,227 ASVs. This means that REFS achieved its highest accuracy ( 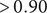 ) with the minimum number of features (16 features), Figure 2A. After running the validation module for the resulting set of features, the Multilayer Perceptron (MLP) classifier had the best performance, with an AUC-ROC of 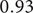 , Figure 2B. In the case of SelectKbest, k = 16, the resulting set of features selected by SelectKbest has 3 in common with those selected by REFS: *Clostridium* (feature 6), *Clostridium sensu stricto 13* (feature 11), and *Dysgonomonas* (feature 15). The classifier with the best performance was Extra Trees with an AUC-ROC of 0.84. According to Šimundić (2009), the AUC-ROC of 0.93 is considered as “*excellent*” diagnostic accuracy.

Additionally to the validation module, we executed MaAsLin2 (Mallick *et al.*, 2021) with a linear model on the resulting reduced set and simultaneously performed a Multivariate Analysis of Variance (MANOVA) model (IBM Corporation, 2011). The results from MaAsLin2 indicate that from the 16 bacterial taxa identified with our methodology, 10 are the most significant: *g.Dialister*, *g.Bifidobacterium*, *g.Clostridium*, *g.Clostridium sensu stricto 13*, *g.Dysgonomonas*, *g.Erysipelatoclostridium*, *g.Clostridium sensu stricto 1*, *g.Clostridium innocuum group*, *g.Streptococcus*, *f.Peptostreptococcaceae.* In the same way, we executed MaAsLin2 on all ASVs in the discovery dataset and used the top 20 as input in the validation module for additional validation. After this process, the resulting AUC-ROC from the classifier with the best performance (Extra Trees) was 0.71. This AUC-ROC is lower compared with the AUC-ROC of REFS (MLP, 0.93) and SelectKbest (Extra Trees, 0.84). The elements at the genus level shared by the set selected by REFS and the top 20 of MaAsLin2 are *Bifidobacterium* (feature 2), *Dialister* (feature 1), *Erysipelatoclostridium* (feature 12), and *Streptococcus* (feature 9). The MANOVA model applied to the microbiome taxa relative abundance matrix demonstrates strong statistical significance, indicating that the collective profiles of the selected taxa have a substantial effect in distinguishing breast milk and infant formula groups (*p* < 0.0001). For detailed information, see Supplementary File 3.

In addition to MANOVA and MaAsLin2, we applied CCREPE (Emma Schwager and Weingart, 2021; Schwager and Huttenhower, 2016) to the selected ASVs to evaluate whether they exhibit meaningful associations rather than being isolated signals. The results confirmed that several pairs have statistically significant relationships after multiple-testing correction, indicating non-random co-occurrence patterns. For example, *g.Clostridium sensu stricto 13* (feature 11) and *g.Erysipelatoclostridium* (feature 13) showed a positive association ( 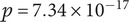 , 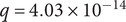 , similarity score = 0.80), *g.Bifidobacterium* (feature 2) and *g.Bifidobacterium* (feature 14) also displayed a positive association ( 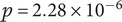 , 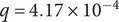 , similarity score = 0.37), and *g.Clostridium* (feature 6) and *g.Klebsiella* (feature 16) showed a negative association ( 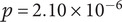 , 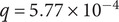 , similarity score = −0.48). These findings confirm that the selected ASVs are embedded in ecological structures, providing biological context and supporting the robustness of the REFS selection. Therefore, the 16 ASVs are not only sufficient for classification but also represent meaningful, non-random relationships that strengthen the interpretability of the results.

**Figure 3.**
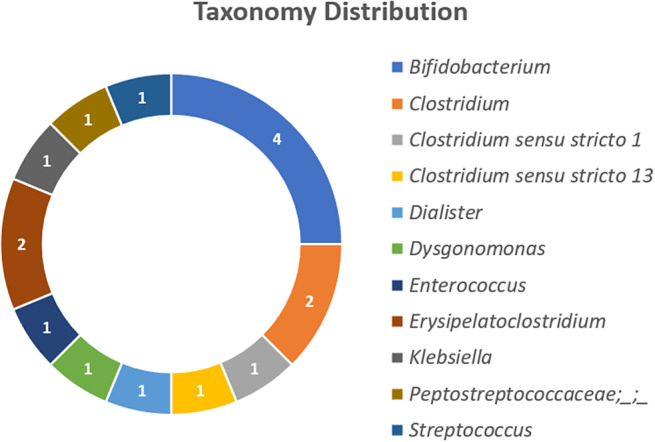
Taxonomy distribution at the genus level. The numbers represent the number of elements contained in each genus-level bacterial taxa.

The addition of BLAST to the taxonomy assignment allowed us to reach the genus level in 15 of 16 taxa, and in some cases, the species level, so the taxonomy distribution is expressed at the genus level. For taxa that did not have resolution at the genus level, it is expressed as *Peptostreptococcaceae;_;_* indicating the family level as the maximum resolution. The visual representation of the taxonomy distribution of the selected ASVs is shown in Figure 3. The taxonomy assignment at all levels, SILVA and BLAST, and the corresponding sequence of the 16 features, as well as which features were found in the testing datasets, are detailed in Supplementary File 1.

### Testing phase

**Figure 4.**
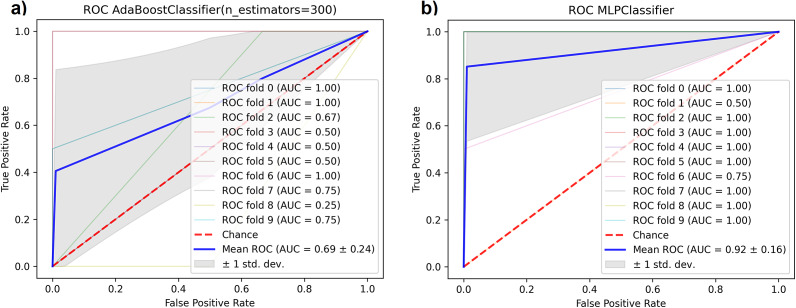
AUC-ROC plots corresponding to the classifier with the best performance for the two testing datasets: (A) AdaBoost for PRJDB7295, (B) MLP for PRJNA562650.

When searching for the selected 16 ASVs in the testing datasets, we found 13 out of 16 ASVs in PRJDB7295 and 12 out of 16 ASVs in PRJNA562650. After running the validation module on the two testing datasets, the best performing classifiers were the AdaBoost classifier for PRJDB7295 with an AUC-ROC of 0.69 and MLP for PRJNA562650 with an AUC-ROC of 0.92, Figure 4. According to Šimundić (2009), the AUC-ROC for PRJDB7295 corresponds to a “*sufficient*” diagnostic accuracy; for PRJNA562650, the diagnostic accuracy is considered as “*excellent.*” Although an AUC-ROC value below 0.7 might be considered marginally acceptable (PRJDB7295), it can still indicate a reasonable discriminatory ability for diagnosis (Mandrekar, 2010). In the case of SelectKbest, we found 5 out of 16 ASVs in PRJDB7295 and 8 out of 16 ASVs in PRJNA562650, with Extra trees being the classifier with the best performance, with an AUC-ROC of 0.58 and 0.79 respectively. In both testing datasets, two of three common features between REFS and SelectKbest were present.

**Figure 5.**
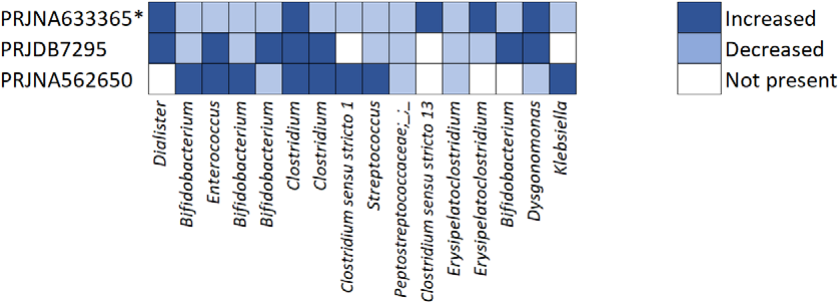
Heatmap for a graphic representation of the relative abundance of the selected taxa (increase or decrease) in breast-milk samples compared to formula samples. Genera are displayed from left to right following the top-to-bottom order as presented in Supplementary File 1.

We conducted an analysis on the relative abundance of the 16 bacterial taxa by calculating the arithmetic average of the relative abundance of each taxon in the breast-milk and formula-fed groups for each dataset. These averages were visualized in a heatmap to provide an intuitive overview of group-level patterns among the identified taxa, as shown in Rojas-Velazquez *et al.* (2025), see Figure 5. The color coding (dark blue = higher in breast milk; light blue = lower) was intended as a descriptive tool to illustrate relative trends, not as a formal statistical test. We acknowledge that microbiome data are compositional and that simple arithmetic means can be misleading for hypothesis testing because of the constant-sum constraint (Gloor *et al.*, 2017). However, in the context of this manuscript, the heatmap serves as a complementary visualization to highlight the behavior of selected taxa across datasets, rather than to infer significance or effect size.

The results indicate that taxa relative abundance varies across datasets, likely due to sequence quality and variations in sequencing equipment, a phenomenon known as the *batch effect* (Rincon *et al.*, 2020), the geographical origins of the datasets, differences in age at which faecal samples were collected, formula composition, and different diets. For example, three of the identified taxa are consistently increased or decreased across all three datasets where they are observed: *Clostridium*, *Peptostreptococcaceae;_;_*, and *Erysipelatoclostridium* (taxa 6, 10, and 12). In contrast with the four *Bifidobacterium* (taxa 2, 4, 5, and 14), which actually show a variation in relative abundance with respect to breastfed versus formula-fed, depending on the dataset. This phenomenon is common in this type of data, so REFS incorporates a nested k-fold cross-validation to mitigate these variances in data and avoid biased results and overfitting (Vabalas *et al.*, 2019). By design, our methodology prioritizes the selection of potential biomarkers that are consistently predictive across different datasets, ensuring that the selected ASVs are robust to the identified technical and biological noise. For example, while regional dietary changes in China, such as the introduction of solid foods after 6 months, influence the composition of the microbiota (Brink *et al.*, 2020), the ensemble-based architecture in a nested k-fold cross-validation of REFS allows the model to keep discriminatory efficiency by focusing on a stable biological signature. Thus, the differences in validation accuracy do not merely reflect data inconsistency but rather demonstrate the model’s ability to generalize findings despite the variability in microbiome datasets.

### MicrobiomeAnalyst

We used MicrobiomeAnalyst (https://microbiomeanalyst.ca) to examine the relationship between the bacterial taxa, diet, and lifestyle using the host-intrinsic taxon functionality. Host-intrinsic taxon analysis revealed that feeding breast milk is associated with a lower abundance of *Peptostreptococcaceae;_;_* and *Erysipelatoclostridium* (taxa 10 and 12), which is consistent with the heatmap results, Figure 5. Additionally, a higher abundance of *Clostridium* and *Enterobacteriaceae* was observed in the host-intrinsic taxon analysis, whereas one of the *Clostridium* (taxa 6) from the 16 selected bacterial taxa showed higher abundance in the breast-fed group in Figure 5. However, one of the *Clostridium* (taxa 7) from the 16 selected bacterial taxa and *Enterobacteriaceae* showed a mixed response in Figure 5. From the analysis, fructose and glucose are associated with an increased abundance of *Clostridium* (taxa 6, 7, and 8), *Klebsiella* (taxa 16), and *Enterococcus* (taxa 3), which is consistent with the heatmap results, Figure 5. Goran *et al.* (2017) show that fructose and lactose are naturally present in breast milk and detected in infants’ gut at 6 months of age.

We also analyzed with the host-intrinsic taxon functionality the selected bacterial taxa to explore their connection with different diseases. The results of the analysis indicated associations with chronic diseases, including cardiovascular diseases, type 2 diabetes, and asthma. In cardiovascular diseases, there was an increased abundance of *Erysipelatoclostridium*, *Bifidobacterium*, *Peptostreptococcaceae;_;_*, and *Streptococcus.* In allergic diseases, such as eczema (allergic dermatitis), a decreased abundance of *Bifidobacterium*, *Dialister*, and *Streptococcus* was observed. For asthma (children under 1 year of age at risk), a decreased abundance of *Bifidobacterium* and *Streptococcus* was also observed.

## Discussion

Based on the results, we observed variations in the relative abundance of the identified bacterial taxa as well as in the AUC-ROC results in the testing datasets compared with discovery. Although the two Chinese datasets have similarities in AUC-ROC, the relative abundance in the testing dataset PRJNA562650 varies with respect to the discovery dataset PRJNA633365. In contrast, the Philippine dataset PRJDB7295 has considerable variation in AUC-ROC, but the relative abundance is similar compared to the discovery dataset. This phenomenon could indicate that geographic regions with different dietary patterns, considering that some babies older than 6 months of age may have included solid food, affect the bacterial composition and should be considered as an additional parameter to be analyzed, as these patterns have a large impact on the microbiome composition during the first year (Brink *et al.*, 2020). Additionally, it can be observed that in comparison with SelectKbest, the features selected by REFS have a better performance considering the AUC-ROC values. Because 16S rRNA sequencing has known taxonomic limitations, we perform an exploratory analysis using a whole-genome metagenomics dataset. This analysis was considered outside the scope of the present study and is therefore not included in the manuscript, but the process and the results are provided in Supplementary File 2.

In addition to evaluating model performance, we analyzed how often each of the 16 final features appeared across the 10 runs of REFS. Five features, Feature 2 (*Bifidobacterium*), Feature 7 (*Clostridium*), Feature 8 (*Clostridium sensu stricto 1*), Feature 10 (*Peptostreptococcaceae*), and Feature 13 (*Erysipelatoclostridium*), were present in all 10 runs, indicating high stability. Two features, Feature 5 (*Bifidobacterium*) and Feature 6 (*Clostridium*), appeared in 9 out of 10 runs, while Feature 1 (*Dialister*) and Feature 9 (*Streptococcus*) appeared in 8 out of 10 runs. Feature 14 (*Bifidobacterium*) was found in 7 runs, and the rest, Features 3, 4, 11, 12, 15, and 16, appeared between 5 and 6 times across the 10 runs. These results show that a subset of the final signature appears consistently, forming a stable core microbial pattern repeatedly identified by REFS, while other features are selected less frequently but still contribute complementary information across runs.

Based on this stability analysis, we analyzed how the selected taxa jointly distinguish breast-fed from formula-fed infants based on the relative abundances of the 16 final features. Breast-fed samples showed higher relative abundance of *Dialister*, *Dysgonomonas*, and certain *Clostridium/Erysipelatoclostridium* ASVs, whereas formula-fed samples showed higher relative abundance of *Klebsiella*, *Streptococcus*, *Enterococcus*, *Peptostreptococcaceae*, and several *Bifidobacterium* and *Clostridium* ASVs. These opposing patterns indicate that the classifiers in the validation module separate feeding groups not through a single organism, but through a coherent combination of ASVs that consistently appear across the resulting features on each high-accuracy REFS run. It is important to note that this interpretation is based on the co-selection patterns observed in the 10 REFS executions and the empirical relative abundance differences in the final result. Some genera contain ASVs associated with opposite feeding groups, highlighting intra-genus heterogeneity and reinforcing that the model captures a community-level signature rather than relying on one taxon.

The genus *Bifidobacterium* (taxa 2, 4, 5, and 14) is more abundant in breast-fed infants and plays a critical role in host homeostasis and immune development, offering protection against allergic diseases (Björkstén *et al.*, 2001), such as asthma (Ronan *et al.*, 2021) and cow’s milk allergy (Francavilla *et al.*, 2012). The study showed a global maturation pattern, with the decline of *Bifidobacterium* after breastfeeding and the expansion of *Faecalibacterium prausnitzii* and *Anaerostipes hadrus* indicating advancing gut microbial maturation. Functional analysis mirrored these taxonomic changes, with central carbohydrate metabolism showing distinct age-linked patterns. For example, *Bifidobacterium breve* utilizes ribokinase to convert ribose into a usable carbon source in early infancy (Fahur Bottino *et al.*, 2025). In contrast, the genus *Clostridium* (taxa 6 and 7), the genus *Clostridium sensu stricto 1* (taxa 8), and the genus *Clostridium sensu stricto 13* (taxa 11) are more abundant in formula-fed infants (Chong *et al.*, 2022), with levels increasing with age (Laursen *et al.*, 2021). This *Clostridiaceae* family is linked to cow’s milk allergy (Hendrickx *et al.*, 2023), while the two genus *Clostridium sensu stricto 1–13* (taxa 8 and 11) are associated with atopic dermatitis (Marrs *et al.*, 2021) and food allergies (Ling *et al.*, 2014). The genus *Streptococcus* (taxa 9), showing a higher abundance in formula-fed infants (Wang *et al.*, 2020), and elevated levels at the family level have been linked to food allergies (Akagawa and Kaneko, 2022). The abundance of these taxa decreases gradually in infant groups from those with cow’s milk allergy (CMA) to cow’s milk sensitization (CMS) to healthy infants (Mennini *et al.*, 2021). The genus *Dialister* (taxa 1) appears to act as a protective factor against food allergies (Akagawa and Kaneko, 2022) and atopic dermatitis (Jin *et al.*, 2023).

*Peptostreptococcaceae;_;_* (taxa 10) is more abundant in formula-fed infants (Azad *et al.*, 2013) and is elevated in those with CMA (Xu *et al.*, 2025). In vitro findings further show that HMO-derived lactate can inhibit strains of *Peptostreptococcaceae*, aligning with the lower abundance observed in exclusively breast-fed (EBF) infants (Huertas-Díaz *et al.*, 2023). In addition, non-EBF infants exhibit a significant increase in the relative abundance of the genus *Erysipelatoclostridium* (taxa 12) compared to EBF infants (Ho *et al.*, 2018). The *Firmicutes/Bacteroidetes* ratio, which evolves from birth to adulthood, undergoes further changes with age (Mariat *et al.*, 2009). The genus *Erysipelatoclostridium* (taxa 13) is more abundant in formula-fed infants (Roggero *et al.*, 2020) and shows increased levels in those diagnosed with allergic conditions (e.g., asthma, food allergies, atopic dermatitis) by 5 years of age (Hoskinson *et al.*, 2023). The genus *Dysgonomonas* (taxa 15) was observed at higher abundance in individuals with food sensitization compared to controls (Savage *et al.*, 2018), although the opposite trend was noted for individuals with cow’s milk protein allergy (Xu *et al.*, 2025). The genus *Klebsiella* (taxa 16) is significantly more abundant in formula-fed infants (Wang *et al.*, 2020).

Ma *et al.* (2020) observed that breast-fed infants showed lower levels of *Clostridioides/Clostridiaceae* across early time points, while formula-fed infants consistently exhibited higher relative abundances of these taxa. *Enterobacteriaceae* were reported as the second most dominant family in all feeding groups, but tended to appear at relatively higher proportions in formula-fed infants. In addition, Ma *et al.* (2020) documented that *Lactobacillus (order Lactobacillales)* was significantly lower in breast-fed infants compared to formula-fed infants at 40 days of age. Li *et al.* (2020) observed that formula-fed and mixed-fed infants exhibited higher relative abundances of *Clostridium sensu stricto 1* and *Erysipelatoclostridium*, both belonging to the *Clostridiaceae/Erysipelotrichaceae* groups, while breast-fed infants showed lower levels of these taxa. *Enterobacteriaceae*-associated genera, particularly *Klebsiella* and *Escherichia–Shigella*, were substantially enriched in mixed-fed and formula-fed infants, whereas breast-fed infants consistently displayed the lowest *Enterobacteriaceae* representation. In contrast, *Lactobacillus (order Lactobacillales)* was reported to be more abundant in breast-fed infants compared with formula-fed and complementary-food-fed infants.

Regarding environmental exposure, factors that are not mentioned, such as household pets and the presence of older siblings, have been linked to early immune conditioning and altered risks for asthma and allergic diseases. Evidence from the KOALA Birth Cohort Study shows that infants with older siblings had slightly higher levels of *Bifidobacteria*, likely reflecting increased microbial exchange within the household. (Penders *et al.*, 2006). Consistent with this, the ALLERGYFLORA study reported lower proportions of *Enterobacteriaceae* (excluding *Escherichia coli)* and *Clostridia*, along with a higher anaerobe-to-facultative anaerobe ratio in these infants, suggesting an accelerated shift towards a more mature, strictly anaerobic gut community (Adlerberth *et al.*, 2007). Recent findings from the HELMi cohort further support this maturation trajectory. Infants with the healthiest gut profiles showed a high initial abundance of *Bifidobacterium*, followed by transitions to *Veillonella* and later to *Faecalibacterium* and *Lachnospiraceae*, reflecting the typical anaerobic succession of early-life microbiota. This progression was associated with environmental and lifestyle factors that enhance microbial exposure, including the presence of siblings, living in a single-family home, and the absence of formula feeding during the first 12 months (Hickman *et al.*, 2024).

In conclusion, we identified specific bacterial taxa differences between breast milk and infant formula and explored associations between these taxa and infant feeding practices. These findings suggest potential differences in gut microbiome composition related to feeding methods and associations with some long-term medical conditions, including food allergies, cardiovascular diseases, and asthma; however, they do not prove cause-and-effect relationships or explain particular functional roles.

Several limitations must be considered. The relatively small sample size may restrict the generalizability of the results. Additionally, confounding factors, including maternal diet, antibiotic exposure, delivery mode, and environmental influences, were not fully controlled and documented during sample acquisition and could have impacted microbiome composition. Another important limitation is the lack of documentation regarding the introduction of solid foods after 6 months of age among the infants from whom samples were collected, which is known to influence gut microbiota development. Furthermore, variability in sampling intervals across datasets introduces additional complexity. It is also important to note that many of these challenges reflect inherent constraints of biomarker discovery and post hoc meta-analysis approaches themselves, rather than limitations unique to this specific method. Finally, the cross-sectional design of this study allows for the identification of associations but cannot determine cause-and-effect relationships.

Future research could address these limitations by adding longitudinal designs, larger and more diverse cohorts, and comprehensive metadata collection. Studying the functional roles of the identified taxa and their interactions with other factors, such as genetics and immune development, will be essential to a better understanding of infant microbiome development and its potential implications for health.

## Supporting information

10.1017/gmb.2026.10020.sm001 Chia Liu et al. supplementary material Chia Liu et al. supplementary material

## Data Availability

The code/scripts to perform experiments are available on GitHub: https://github.com/steppenwolf0/MicrobiomeREFS. The results from the DADA2 amplicon workflow and REFS analysis, along with data from the exploratory Whole Genome Metagenomics experiment and supporting scripts, are available on GitHub: https://github.com/EDavidRojas-Velazquez/breastfed_versus_formula-fed_data-scripts.git

## Supplementary material

The supplementary material for this article can be found at http://doi.org/10.1017/gmb.2026.10020.
