# Supplementary material for: Machine learning identifies differences between breast milk and formula in the gut microbiome
Source: Gut Microbiome (Camb). 2026 May 8;7:e7. doi: 10.1017/gmb.2026.10020 (PMC13161719; doi:10.1017/gmb.2026.10020)
Supplement: Chia Liu et al. supplementary material [file S2632289726100206sup001.zip › Supplementary file 2.docx]

**Exploratory Whole-Genome Metagenomics Analysis of Infant Gut Microbiome**

We performed an analysis of a whole genome metagenomic dataset (accession number PRJNA542703) consisting of 30 paired-end samples distributed in 20 breast-fed samples (labeled as 0) and 10 formula-fed samples (labeled as 1). The dataset was collected from fecal samples of infants in the United States. The methodology applied is different from the one used in this manuscript. We performed the quality control process using Trimmomatic (Bolger, Lohse, & Usadel, 2014) by using the default parameters such as Phred quality established in 33 as indicated in the tutorial (Usadel Lab, n.d.). For taxonomy assignment we used Kraken2 (Wood, Lu, & Langmead, 2019). As with Trimmomatic, we use kraken2 with the default parameters described in the tutorial (Wood, n.d.). To generate the final taxonomy report and the corresponding relative abundance estimation, we used Bracken (Lu, Breitwieser, Thielen, & Salzberg, 2017) with the default parameters to generate the report at species level described in the tutorial (Lu, n.d.).

Once we have the metagenome species taxonomic level, we compared it with the taxonomic list we generated with our methodology, made a match between the different taxonomic levels reached with 16s rRNA and obtained the following 12 species candidates: *Bifidobacterium adolescentis, Bifidobacterium longum, Bifidobacterium pseudolongum, Bifidobacterium breve, Bifidobacterium animalis, Bifidobacterium bifidum, Bifidobacterium thermacidophilum, Streptococcus luteciae, Streptococcus alactolyticus, Streptococcus infantis, Streptococcus anginosus, Streptococcus equi.* After executing a validation process using several classifiers, we obtained an AUC-ROC of 0.97 with the GradientBoosting classifier, which was the one who showed the best performance, Fig. 1a. In the original study, we obtained AUC-ROC of 0.69 (AdaBoost classifier for PRJDB7295) and 0.92 (MLP classifier for PRJNA562650). In comparison with the metagenome analysis, the same classifiers showed AUC-ROC of 0.92 for the AdaBoost classifier (Fig. 1b) and 0.7 for MLP classifier (Fig. 1.c), presenting a positive tendency between technologies.


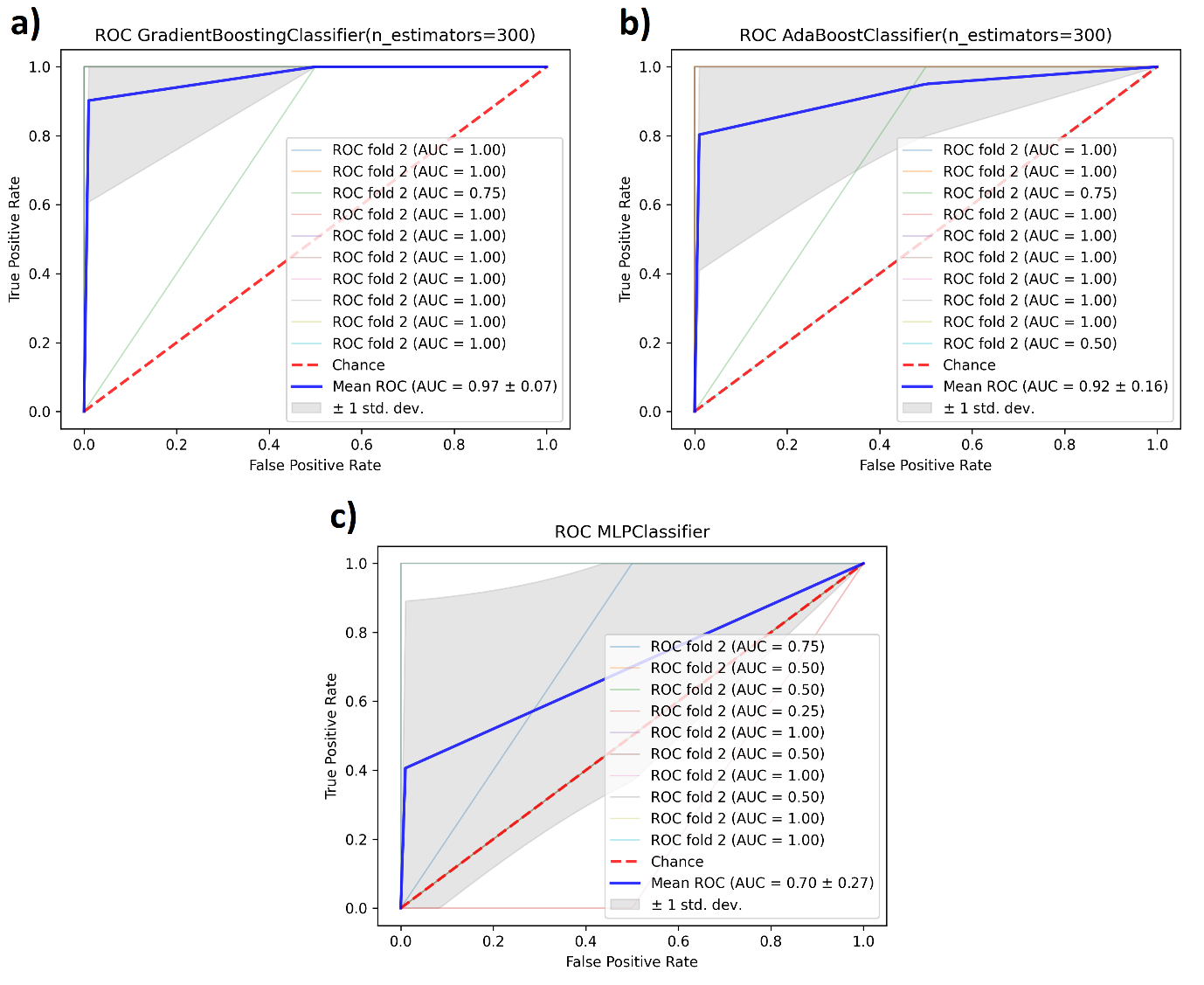


**Fig. 1.** AUC-ROC plots for the metagenome experiments: a) the algorithm with the best performance GradientBoosting Classifier, b) AdaBoost classifier, c) MLP Classifier.

**References**

Bolger, A. M., Lohse, M., & Usadel, B. (2014). Trimmomatic: A flexible trimmer for Illumina Sequence Data. Bioinformatics, btu170.

Lu, J. (n.d.). Bracken. GitHub. Retrieved December 1, 2025, from https://github.com/jenniferlu717/Bracken

Lu, J., Breitwieser, F. P., Thielen, P., & Salzberg, S. L. (2017). Bracken: estimating species abundance in metagenomics data. PeerJ Computer Science, 3, e104.

Usadel Lab. (n.d.). Trimmomatic: A flexible read trimming tool for Illumina NGS data. Retrieved December 1, 2025, from http://www.usadellab.org/cms/?page=trimmomatic.

Wood, D. (n.d.). Kraken 2 manual. GitHub. Retrieved December 1, 2025, from https://github.com/DerrickWood/kraken2/wiki/Manual.

Wood, D. E., Lu, J., & Langmead, B. (2019). Improved metagenomic analysis with Kraken 2. Genome biology, 20(1), 257.
