# Supplementary material for: Machine learning identifies differences between breast milk and formula in the gut microbiome
Source: Gut Microbiome (Camb). 2026 May 8;7:e7. doi: 10.1017/gmb.2026.10020 (PMC13161719; doi:10.1017/gmb.2026.10020)
Supplement: Chia Liu et al. supplementary material [file S2632289726100206sup001.zip › Supplementary file 3.docx]

**MANOVA**

In this study, the multivariate analysis of variance (MANOVA) follows the standard linear model expressed as Y=XB+E, where Y represents the matrix of dependent variables (the multivariate features), X is the design matrix constructed from the predictor variable. The matrix B contains the estimated regression coefficients describing how the predictor influences each dependent variable, while E represents the residual or error matrix capturing the unexplained variation in the model. MANOVA evaluates whether variation in B across groups is significantly greater than the within‑group variation represented by E. This analysis was performed using the MANOVA implementation from the *statsmodels* Python library (Seabold & Perktold, 2010). MANOVA provides a useful multivariate view of the data, but it does not account for particular properties of microbiome datasets such as sparsity and compositionality. Because MANOVA assumes unconstrained continuous variables in Euclidean space, it cannot model zero inflation or the dependencies created by the constant‑sum structure. Therefore, we apply MANOVA only as an exploratory multivariate tool and interpret its results with caution.

The MANOVA model applied to the microbiome taxa relative abundance matrix demonstrates strong statistical significance, indicating that the collective profiles of the selected taxa have a substantial effect in distinguishing breast milk and infant formula groups. This is supported by several multivariate test statistics: Wilks’ Lambda = 0.1101 (Num DF = 16, Den DF = 49), suggesting that a large proportion of the variance in group labels is explained by the taxa data; Pillai’s Trace = 0.8899, evidencing a robust multivariate effect; and Hotelling–Lawley Trace = 8.0802 as well as Roy’s Greatest Root = 8.0802, further confirming the strength of the model. The overall F = 24.7456 with p < 0.0001 indicates that the observed group differences are highly unlikely to be due to chance.

**Reference**

Seabold, S., & Perktold, J. (2010). Statsmodels: econometric and statistical modeling with python. *SciPy*, *7*(1), 92-96.
